# Green-synthesized zinc oxide nanoparticles and walnut biochar synergistically mitigate soil salinity and improve maize stress physiology

**DOI:** 10.3389/fpls.2026.1788236

**Published:** 2026-03-13

**Authors:** Sangar Khan, Jaweriah Naeem, Aansa Rukya Saleem, Fiza Sarwar, Asma Jamil, Habib Ullah, Zepeng Rao, Abubakr M. Idris, Waqar-Un Nisa

**Affiliations:** 1 Department of Geography and Spatial Information Techniques, Ningbo University, Ningbo, China; 2 Zhejiang-Germany Joint Laboratory on Remote Sensing of Coastal Ecosystem, Ningbo University, Ningbo, China; 3 Department of Earth and Environmental Sciences, Bahria School of Engineering and Applied Science, Bahria University, Islamabad, Pakistan; 4 Department of Environmental Science, Zhejiang University, Hangzhou, China; 5 Innovation Center of Yangtze River Delta, Zhejiang University, Jiashan, China; 6 Department of Chemistry, College of Science, King Khalid University, Abha, Saudi Arabia; 7 Research Center for Advanced Materials Science (RCAMS), King Khalid University, Abha, Saudi Arabia; 8 Center for Interdisciplinary Research in Basic Sciences (SA-CIRBS), International Islamic University, Islamabad, Pakistan

**Keywords:** antioxidant enzymes, biochar, green synthesis, maize, soil salinity, sustainable agriculture, zinc oxide nanoparticles

## Abstract

**Introduction:**

Soil salinization constrains maize productivity by elevating osmotic stress, disturbing nutrient homeostasis, and intensifying oxidative injury. In this study, we developed a green nano-biochar approach by synthesizing zinc oxide nanoparticles (ZnO NPs) from *Coriandrum sativum* leaf extract and combining them with walnut shell biochar (BC) to form a composite soil amendment, which may increase the nutrients (N, P, K) and increase plant antioxidant defenses in saline soil.

**Material and methods:**

ZnO NPs were verified as crystalline ZnO with wurtzite structure and nanoscale morphology using Scanning Electron Microscopy – Energy Dispersive X-ray Spectroscopy (SEM-EDS), Fourier Transform Infrared (FTIR), and X-ray Diffraction (XRD). A factorial pot experiment (15 days) was conducted in moderately (S-1; 4–5 dS m^-1^) and highly saline (S-2; 9–10 dS m^-1^) soils to compare ZnO NPs, BC, and composite against the unamended control.

**Results and discussion:**

Additionally, maize plants treated with the composite demonstrated improved morphological traits, including a 43% increase in shoot, 41% higher total chlorophyll content, and a 28% increase in antioxidant enzyme activity. Stress diagnostics further showed improved membrane stability, with lower reactive oxygen species (ROS) burdens and reduced peroxidation and EC under saline conditions. Across both salinity levels, the composite consistently outperformed single amendments, underscoring the value of integrating ionic buffering with micronutrient delivery. By coupling plant extract synthesis and valorization of walnut residues, this study offers a resource-efficient alternative to conventional salinity management and offers a promising approach for potential crop productivity and soil health in saline-affected agricultural lands. However, studies should be done on the large scale in experimental fields.

## 1 Introduction

The global agricultural sector and crop production are seriously threatened by the accumulation of salt in the soil. Soil salinity is a widespread environmental and agricultural challenge affecting nearly 10% of global arable land (Syed et al., 2021; Atta et al., 2023). Soil is a fundamental natural resource that supports ecosystem functioning, material and energy cycling, and agricultural productivity, while also playing a key role in pollutant attenuation and water regulation (Meng et al., 2025). High concentrations of soluble salts disrupt nutrient uptake, cause ion toxicity, and generate osmotic stress that limits plant growth (Pandit et al., 2024). High sodium and chloride ions displace essential nutrients from soil colloids, reducing phosphorus solubility and limiting nutrient diffusion to roots. Additionally, climate change is expected to intensify salinization through seawater intrusion, rising temperature, and increased evapotranspiration (Ullah et al., 2021). Moreover, the intensification of climate change driven by increasing greenhouse gas emissions and extreme weather events is further exacerbating environmental stresses on agricultural systems, thereby threatening crop productivity and global food security (Lin et al., 2024). In Pakistan, over 50% of irrigated lands face salinity-related productivity losses (Syed et al., 2021). Maintaining the health of the land and guaranteeing a steady supply of food depend on agriculture (Haq et al., 2023). The issue of soil salinity in Pakistan can be attributed to both natural processes and human-induced factors. Natural processes such as the gradual weathering and erosion of rocks, the intrusion of seawater in coastal areas, and the high rates of evaporation in the arid climate all contribute to the buildup of salts in the soil (Sagar et al., 2021). However, the problem has been exacerbated by human activities, such as the over-exploitation of groundwater for irrigation, the use of poor-quality water for crop production, and the lack of proper drainage systems to mitigate the accumulation of salts. As a result of these factors, it is estimated that up to 50% of the irrigated land in Pakistan has become saline, leading to a critical loss of yield profitability every year (Zahra et al., 2024). Conventional techniques of managing salinity mainly focused on salt accumulation in the root zone. These techniques include the leaching of excessive salts with the use of good-quality irrigation water, an adequate drainage system, the application of chemicals (gypsum), improved irrigation practices to control salt movement, and water tables. Soil management practices such as land leveling, organic matter incorporation, and the use of salt-tolerant varieties are mostly used. These conventional approaches to treating soil salinity are often slow, costly, or environmentally harmful (Chen et al., 2025).

Nanoparticles (NPs) have garnered considerable attention in environmental cleanup and remediation due to their high surface area and reactivity, offering promising potential in alleviating salinity stress through improved nutrient delivery, enhanced ROS regulation, and soil remediation (Yadav et al., 2023). Agricultural bioremediation, such as nano-biotechnology, aids in restoring the soil’s natural state by resolving environmental issues using sustainable remediation technology. It’s an interesting phenomenon to think about how hazardous elements can be removed from agricultural soil and turned into a sustainable resource through nanotechnology (Prasad et al., 2017). Nano-enabled soil remediation techniques have shown promise in addressing the underlying causes of salinization, such as the collection of toxic ions and the depletion of key nutrients (Yadav et al., 2023). Another benefit is the further modifications to their size-related chemical, physical, and biological characteristics (Khan, 2019). According to several recent research studies, nanoparticles are among the most effective substitutes for currently employed dubious methods of addressing abiotic stressors in plants (Mishra et al., 2025). In the current decade, zinc oxide nanoparticles (ZnO NPs) have been used extensively in agricultural production. It has been reported that ZnO NPs enhance antioxidant activity, nutrient uptake, and stress tolerance in crops such as beans, rice, and wheat (Gupta et al., 2024). Focused research has been conducted on ZnO NPs due to their distinctive electrical, optical, mechanical, magnetic, and chemical properties, which differentiate them from their bulk counterparts (Parihar et al., 2018). The work of Thounaojam et al. (2021) demonstrated that they can be applied as nano fertilizers, nano pesticides, nano biosensors, and remediating contaminated soils (Thounaojam et al., 2021). Additionally, applying NPs enhances the expression of stress-tolerant genes and proteins in plants, making them more resistant to abiotic and biotic stresses. The exceptional properties of NPs make them ideal for promoting sustainable agriculture. ZnO NPs have been found to reduce salinity stress in common beans. Through a comprehensive examination covering physiological, biochemical, and nutritional parameters, it has been demonstrated that ZnO NPs are effective in mitigating the negative effects of salinity stress on bean plants. ZnO NPs can modify antioxidant defense mechanisms, thus reducing the severity of oxidative damage and promoting the growth of plants under salinity stress (Mogazy and Hanafy, 2022; Gupta et al., 2024).

In other soil remediation methods, it has been demonstrated that reclaiming salt-damaged soil using biochar (BC) as a soil supplement improves the salinity-affected croplands. In addition to soil improvement, biochar has also been widely applied in industrial and agricultural wastewater treatment because of its high surface area and adsorption capacity for organic and inorganic pollutants (Zhou et al., 2026). Biochar, a carbon-rich byproduct of biomass pyrolysis, enhances soil characteristics by augmenting water retention, cation exchange capacity (CEC), organic matter content (OMC), and nutrient stability (Da Silva Medeiros et al., 2025; Ali et al., 2025). Walnut BC is especially beneficial due to its porosity and mineral-rich structure. It has gained interest in agriculture for improving degraded soil and promoting plant growth by enhancing soil properties and enzymatic activities. In comparison to the control, plants grown in salinity without biochar had reduced chlorophyll content by 9.7%, 17%, and 30%. The addition of biochar improved chlorophyll levels. Under typical conditions, 2% biochar increased chlorophyll content by 4%, and 1% BC increased it by 7% (Akhtar et al., 2015). Since the development of nanotechnology, nano-biochar has emerged as a promising tool for removing pollutants and promoting sustainable agriculture. Made from specific raw materials through pyrolysis at different temperatures, nano-BC enhances crop growth and improves soil health. Its tiny particles and nanostructure help the soil breathe better, retain water, and reduce evapotranspiration. This keeps the soil environment healthy for plants by reducing salt ions in areas with high salinity (Rajput and Minkina, 2025). Research on rice straw-derived BNPs showed that BC amendments improve the physical, chemical, and biological properties of saline soils, ultimately enhancing soil quality and tomato plant growth (Tao et al., 2023). Furthermore, a field experiment explored the potential of BNPs to mitigate the effects of soil salinity in more realistic growth conditions. The experiment involved transplanting tomato plants into soil supplemented with 0.2% NaCl (w/w) and varying concentrations of BNPs (Zhang et al., 2024).

Maize is one of the leading cereals along with wheat and rice. It plays a vital role in global food security by providing 30% food calories to more than 57% population of the globe, nutrition, and economic development (Salam et al., 2022). It covers 200 Mha and produces over 1000 million tons globally (Liu, 2021). Use of ZnO NPs and biochar (BC) in mitigating stresses is a very interesting and new field. While the previous studies tested ZnO NPs alone or biochar alone, this research used a green-synthesized ZnO NP + BC composite for salinity mitigation. The study hypothesizes that ZnO NPs integrated with nano BC application may increase the nutrients (N, P, K) and increase plant antioxidant defenses in saline soil. The objectives of this study were 1) to synthesize the nano ZnO+BC composite, 2) to evaluate its effectiveness in increasing the maize plant response to saline soil, and 3) to increase the concentration of N, P, and K in saline soil.

## 2 Materials and methods

### 2.1 Sample area and sampling

A soil sample was collected (0–15 cm) from Firoza in Rahim Yar Khan (28° 45’ 0” North, 70° 49’ 0” East), a warm desert region with low rainfall and high evaporation. The soil texture ranges from sandy loam to loam. This salt-stressed sample from agricultural land has high water retention, making it less suitable for plant growth due to salt. A second sample from Islamabad, ideal for plant growth, was later subjected to salt stress with a target salinity conductivity of< 8 dS m^-1^ to evaluate treatment effectiveness in high-saline soil. The soil samples were classified into the moderately saline soil (S-1; EC 4–5 dS m^-1^) and the artificially saline soil (S-2; EC 9–10 dS m^-1^). This was achieved by gradually adding NaCl solution to avoid the osmotic shock. Both soils were air-dried, sieved (5 mm), and homogenized. The samples were stored in Ziplock bags at 5°C until needed for experimentation. The water holding capacity of the samples was determined before the experiment by calculating the mass of water retained per mass of dry soil. The 50 g soil was saturated with the deionized water (DI) for 4 hours. The saturated soil was suspended in a funnel (having filter paper), and the soil was drained for 78 hours (Khan et al., 2022). After knowing the water holding capacity, the soil samples were pre-incubated for 7 days. A pot experiment was performed to evaluate soil parameters and plant growth. The soil was sieved through a 5 mm mesh sieve to eliminate rocks and debris, resulting in fine soil, which was then distributed among the pots. The setup included both S-1 and S-2 soils.

### 2.2 ZnO NPs synthesis and characterization

The ZnO NPs were synthesized with the leaf extract of coriander (*Coriandrum sativum*). Briefly, fresh leaves were collected, shredded, and oven-dried at 50°C for 30–45 minutes to remove moisture. They were then washed with deionized water to eliminate dust. To create the extract, 50 grams of the dried leaves were added to a 500 ml glass beaker with 200 ml of DI water. The mixture was heated until it turned dark yellow. After cooling, the extract was filtered using Whatman filter paper (No. 40). The resulting dark yellow to green extract will serve as a reducing agent (Ukidave and Ingale, 2022). To prepare the solution, 1 g of zinc acetate


$$\left(Zn{\left(CH_{3}CO_{2}\right)}_{2}\right)$$

were dissolved in 50 ml of distilled water and stirred using a magnetic stirrer. Gradually add 1 ml of coriander leaf extract while stirring. The concentration of zinc acetate was adjusted according to the requirements and mixed for 2 hours.

The pH was checked using a pH meter, and 2 M NaOH was added dropwise until the pH reached 12. The mixture was then shaken for another hour (Sabir et al., 2014). Centrifugation of the mixture was performed at 3000 rpm for 40 minutes, and ZnO NPs were collected in a Petri dish. Finally, NPs were placed in an oven for drying at 60 to 80°C, and crushing was performed with a pestle and mortar. To understand the nature of ZnO NPs, characterization was done by XRD, TEM, SEM, FTIR, and EDX analysis. The crystalline structure of ZnO NPs was predicted by using X-ray diffraction spectra (XRD). In the spectra, Rigaku (D/max 2400/pc) with Cu (λKα = 1.5406Å) was used as a source, and the data were recorded with 2^1^ ranging from 20-80° (Azhar et al., 2020). The particle size and surface morphology of the ZnO NPs were assessed through TEM (JEM-1230, JEOL, Akishima, Japan) and SEM (TM-1000, Hitachi, Japan).

### 2.3 Nanocomposite biochar production

BC from walnut was prepared. For this purpose, raw nutshell material was collected, air-dried, chopped into small pieces, and placed in an oven at 105°C for 1 hour to remove the remaining moisture. The dried material was pyrolyzed at 450°C for 20 minutes, then cooled and stored in an airtight container with silica gel. To ensure uniform distribution, the BC was sieved to a 0.5mm size and ground into a fine powder. The scanning electron microscope was used to capture images of the biochar. The pH, EC, Ash contents, N, and P were analyzed according to their standard protocols. These biopolymers are integrated with organic or inorganic materials (including metals and metal oxides) at the nanometer scale to enhance specific properties of the resulting composite materials (Ali et al., 2021). In each treatment, 0.7g of ZnO NPs was incorporated with 700g of BC. First, the ZnO NPs solution was poured into the biochar and left for 24 hours. Extra moisture was removed with the help of filter paper. The formed nanocomposite was applied as a separate treatment, with the concentration varying according to the soil’s weight.

### 2.4 Experimental design and pot incubation

A factorial experiment (2 salinity levels × 4 treatments) was conducted with 3 replicates per treatment. Maize is moderately sensitive to salt stress, and 100 mM of NaCl reduced the crop yield by up to 50% (Cao et al., 2019). The experiment used two soil types: S-1, moderately saline soil from barren agricultural land with an electrical conductivity (EC) of 4–5 dS m^-1^, and S-2, artificially created saline soil with an EC of 9–10 dS m^-1^. 42 pots received ZnO NPs and biochar treatments, applied separately and in combination. BC was added at 2% (100 g per kg of soil) (Zheng et al., 2013), while ZnO NPs were applied at 0.7 g for 7 kg of soil (Adil et al., 2022). NPs were mixed in the soil at the time of sowing. Readings were taken from Day 1 to Day 15, with observations made before and after treatment, and results were categorized for Soil-1 (S-1) and Soil-2 (S-2) based on the treatments and days. Seven treatments were created: 1- S (Control, no amendments); 2-Soil and ZnO NPs (S + NP); 3-Soil and Biochar (S + BC); 4-Soil and Nanobio composite (S + NP + BC); 5-Plant + NP (P + NP); 6-Plant + BC (P + BC); 7-Combined application (P + NP + BC). The incubation experiment was performed in pots of area 20 cm × 18 cm. The pots were filled with soil and amendments as described in the experimental design. Phosphorus (P) was applied as a basal dose (1.2 g KH_2_PO_4_) to all the pots. The water-holding capacity of the soils was maintained at up to 50%. The maize (local hybrid YH-1898) was planted in pots for 15 days at 28°C during the day and 20°C at night, with a 14-hour photoperiod and 60% humidity.

### 2.5 Soil chemical analysis

The 5 g of soil sample was collected from the pot. The soil was thoroughly combined with 50 mL of distilled water. The sample was stirred gradually until it was thoroughly mixed, and then it was filtered. The sample was tested with a multimeter to check the pH (1g: 5mL) of the respective samples. The nitrogen content in the soil was measured by the nitrate method (Khan et al., 2025). The prepared sample was measured with a UV spectrophotometer at 220 nm. The values were further calculated using the method described by (Allende-Montalbán et al., 2024).

Phosphorus content in the soil was measured with the help of a spectrophotometer by using a wavelength of 882 nm. For this purpose, a 5 N NaOH solution was prepared by dissolving 200 g NaOH in distilled water. 0.5 M NaHCO_3_ solution was also prepared, and pH was adjusted to 8.5 with 5 N NaOH and 5 N H_2_SO_4_. Special reagents included p-nitrophenol indicator, Reagent-A (ammonium heptamolybdate and antimony potassium tartrate in sulfuric acid), and Reagent-B (L-Ascorbic acid mixed with Reagent-A) were also used. A standard stock solution for phosphate (500 ppm P) was prepared from dried KH_2_PO_4_. Standard working solutions with known concentrations of P (1, 2, 3, 4, and 5 ppm P) were created by diluting the stock solution. These components are utilized sequentially to measure phosphate levels via UV spectrophotometer analysis (Qazi, 2021). The potassium in the soil was measured by mixing 5 g of soil with 50 ml of distilled water (Estefan et al., 2013). The prepared sample’s absorbance was measured at 766 nm. The obtained absorbance was calculated based on the curve created using the potassium in the soil. The organic matter (OM) was calculated using the loss on ignition (LOI) method (Robertson, 2011). For this purpose, oven-dried at 100 °C for 11 hours10g of the soil sample was used. Weight loss was considered to be the removal of moisture content from the soil. Then the sample was heated in the furnace at 400 °C. The sample was kept in the stove for 2 hours to remove the organic content of the soil. Weight loss after ignition was considered to be a measure of organic matter content.

### 2.6 Plant parameters analysis

#### 2.6.1 Measurement of chlorophyll contents

Fresh leaf samples of maize were harvested from all the treatment groups and chopped into small pieces. Then dipped in 85% acetone for 24 hours. Afterwards, the content of total chlorophyll, chlorophyll *a*, and *b* was quantified using a spectrophotometer at different wavelengths (663, 645, and 470 nm) (Adeel Zafar et al., 2021).

#### 2.6.2 Measurement of antioxidants and proline

For the determination of Proline, 250–500 mg of fresh leaf tissue was homogenized in a cold pestle and mortar by adding 5 mL of 3% sulphosalicylic acid. Whatman Filter Paper No. 2 was used to filter the homogenate produced. To create a colored complex, 2 mL of the filtered sample was then combined with 2 mL of acid-ninhydrin, along with glacial acetic acid. The mixture was then agitated and incubated at 100°C for an hour in a water bath. The test tubes were then chilled in an ice bath. In each sample, 4 mL of toluene was added, then the mixture was vortexed to separate the aqueous and organic phases, allowing for the extraction of the pink layer formed. The absorbance of the prepared sample was recorded at 520 nm in a UV spectrophotometer (Tamayo and Bonjoch, 2001).

To analyze the peroxidase enzyme in the plant, leaves were crushed using a pestle and mortar, adding phosphate buffer until a pH of 7 was achieved (Senthilkumar et al., 2020). The prepared sample was then centrifuged at 2400 rpm for 15 minutes, and the liquid sample was separated. The 0.1 substrate, 4-methyl pyrocatechol, was added to 1 mL of plant extract. Subsequently, 2 mL of phosphate buffer and 2 mL of hydrogen peroxide were added to the sample. The sample absorbance was checked at 420 nm in a UV spectrophotometer. Peroxidase values were further calculated by using the formula given with 2.8 mM^-1^cm^-1^ as the coefficient.


$$Activity\;\left(\frac{U}{mL}\right)=\left(\frac{\Delta Abs}{\Delta t}\right)\times \;\left(\frac{1}{\epsilon }\right)\times \left(\frac{total\;volume}{Enzyme\;volume}\right)$$

For the catalase measurements, the leaves were crushed with the 0.1 M phosphate buffer in the cold pestle and mortar. The sample was then centrifuged at 1200 rpm for 24 minutes. After that, the dense particles were settled at the bottom of the Eppendorf, whereas the liquid layer was collected. In the collected sample, hydrogen peroxide and phosphate buffer were added to get a volume of 3ml. The absorbance was checked at 240nm in a UV spectrophotometer (Sarker and Oba, 2018).

#### 2.6.3 Quantification of electrolyte leakage, MDA, and H_2_O_2_

For the measurements of electrolyte leakage (EL), leaf samples were divided into small pieces of 1 cm, followed by washing with ddH_2_O. Incubation at room temperature was carried out for 24 hours. First reading (EC1) was taken. The same samples were placed at 120 °C for 20 minutes, and after cooling second reading (EC2) was taken.


$$EL\;\left(\%\right)=\frac{EC1}{EC2}\;\times 100$$

MDA amount was calculated by following the previously published protocol (Thomas et al., 2004). For this purpose, a fresh 1-gram sample was used, and values were calculated by using the following formulae:


$$Concentration\;of\;MDA\;\left(mM\right)=\left(A_{532}-A_{600}\right)/155$$

For the quantification of H_2_O_2_, fresh samples were homogenized in 0.1% chilled trichloroacetic acid, followed by centrifugation at 12000 rpm for 15 minutes at 4°C. 10mM Potassium phosphate buffer (pH 7.0) and 1 M potassium iodide solution were added. Readings were taken at 390 nm using a spectrophotometer.

### 2.7 Measurement of gas-exchange traits

The gas exchange traits (Pn, Gs, and Ci) were measured as previously described by (Holá et al., 2010). In detail portable gas-exchange system (LCpro+, ADC Bioscientific Ltd, Hoddesdon, UK) was used during the morning time (8:30 am - 11:30 am). In the chamber, 650 mmol m^-2^s^-1^ was PAR, CO2 was 550 cm3 m-3, the temperature was set to 25°C, while the air flow was 205 ± 30 mmol s^-1^. Readings were taken after 10 minutes of steady state conditions.

### 2.8 Statistical analysis

The current experiment was conducted in three replicates. Factorial analysis was used to assess the variation among the treatments in Statistix (8.0) software. Data was represented by mean values and standard error (SE), while the significance was checked by Fisher’s Least Significant Differences (LSD).

## 3 Results

### 3.1 ZnO NPs and biochar analysis

**Figure 1:**
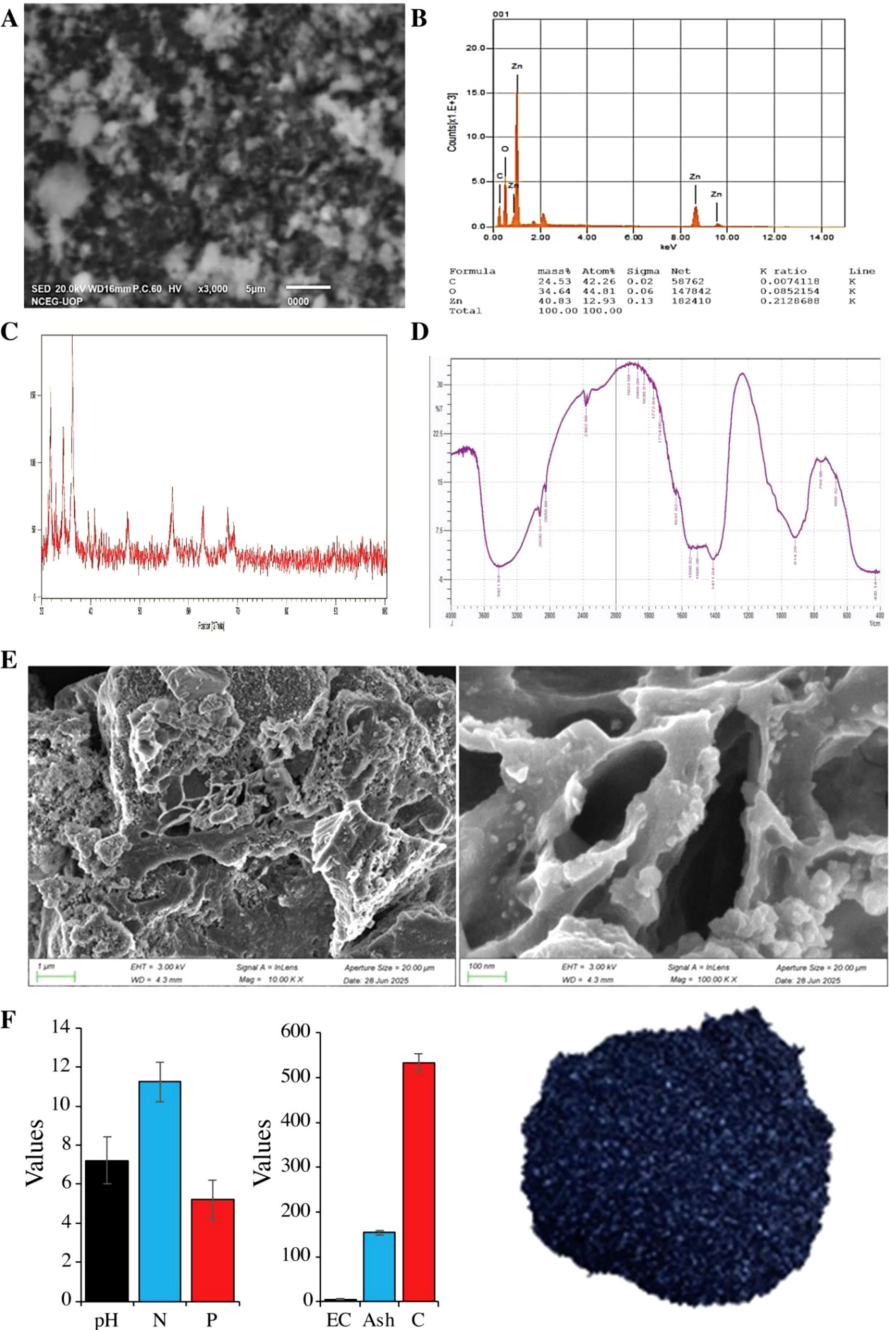
Physiochemical characterization of green-synthesized zinc oxide (ZnO) nanoparticles and walnut biochar. The scanning electron microscopy (SEM) images and corresponding energy dispersive X-ray spectroscopy (EDS) spectra showing the morphology and elemental composition of ZnO nanoparticles **(A–C)**. The Fourier-transform infrared spectroscopy (FTIR) spectrum of ZnO NPs indicating characteristics functional groups and Zn-O bond vibrations **(D)**. SEM image illustrating the porous surface morphology **(E)**, and physiochemical properties of walnut biochar including the pH (potential of hydrogen); N (total nitrogen); P (available phosphorous); EC (electrical conductivity); Ash, and C (total carbon) contents of biochar **(F)**.

The average particle size of the synthesized NPs was recorded as 53.76 nm. The image, taken at 3000x magnification, shows ZnO NPs with a 5 µm scale bar indicating nanoscale agglomerates (**Figure 1A**). EDS (Energy-dispersive spectroscopy) was used to confirm the synthesis of ZnO NPs further. For this purpose, corresponding peaks were measured. It confirms a composition of 40% zinc, 34% oxygen, and 24% carbon, validating the presence of ZnO (**Figures 1B, C**). The results support the effective production of ZnO NPs. For recording of NPs spectra, FTIR spectroscopy was used (**Figure 1D**), which affects their optical and electrical properties. For this purpose, a frequency range of 400–4000 cm^-1^ was utilized. The FTIR analysis of vibrational frequencies associated with bonds identifies the functional groups on the surface of the NPs. By examining the interaction between the capping agent of ZnO NPs and the FTIR data, the probable bonds in the biomolecule responsible for the capping peaks and the stability of the metal NPs synthesized with *C. sativum* leaf extract were established. Key findings include a broad absorption band at 3421.83 cm^-1^ for O-H stretching (hydroxyl groups), peaks at 2920.32 cm^-1^ and 2850.68 cm^-1^ for C-H stretching, and 1744.08 cm^-1^ for C=O stretching (carbonyl groups). Sharp peaks at 914.29 cm^-1^ and 759.98 cm^-1^ confirm the presence of Zn-O bonds, indicating successful synthesis and the presence of functional groups that enhance stability and dispersibility. The diffraction pattern of ZnO NPs exhibits clear peaks at specific 2θ values between 30° and 100°, corresponding to different crystal planes and indicating the hexagonal wurtzite structure of ZnO (**Figure 1E**). Specifically, the prominent peaks are observed at approximately 31.7°, 34.4°, 36.3°, 47.6°, 56.7°, and 62.8°, corresponding to the (100), (002), (101), (102), (110), and (103) planes of ZnO, indicate the crystalline nature and good crystallinity in the ZnO NPs. The pH of the biochar was 7.22, with N and P contents of 11.25 and 5.21 g kg^-1^, respectively. The EC of the biochar was 5.40 dS m^-1^, the ash contents were 154.21, and the C contents were 534 g kg^-1^ (**Figure 1F**).

### 3.2 Soil pH, salinity, and organic matter contents

**Figure 2:**
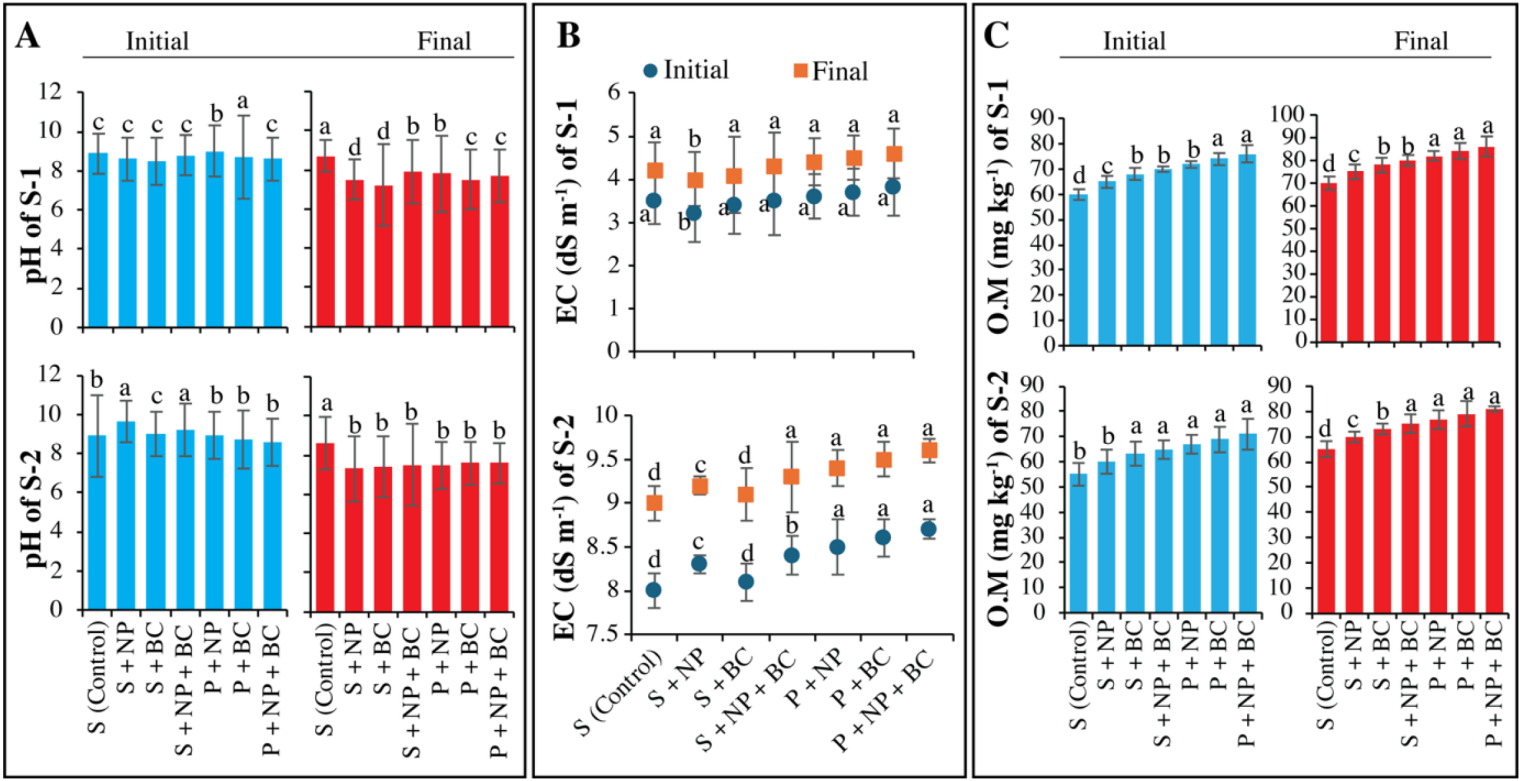
Effect of zinc oxide nanoparticles and walnut biochar on soil chemical properties under natural and artificial salinity conditions. Soil pH **(A)**, electrical conductivity [EC, **(B)**], and organic matter **(C)** in natural saline soil (S-1) and artificially induced saline soil (S-2). The S-1 and S-2 final represent the values after 15 days of treatment application. Error bars indicate the standard error (SE) of the mean (n = 3). Columns sharing the same lowercase letter are not significantly different (P ≤ 0.05).

The control groups S-1 and S-2 exhibited only slight pH reductions (8.83 and 8.74), showing poor salinity suppression (**Figure 2A**). In contrast, ZnO NPs (S+NP) significantly lowered pH from 8.61 to 7.58 in S-1 and 9.67 to 7.25 in S-2 after 15 days, especially effective in the high salinity condition (S-2). The P+NP treatment also reduced pH from 8.95 to 7.82 in S-1 and from 8.81 to 7.49 in S-2. Additionally, the S+BC treatment lowered pH from 8.56 to 7.28 in S-1 and from 8.94 to 7.21 in S-2, highlighting biochar’s effectiveness. The P+BC treatment decreased pH from 8.71 to 7.58 across both soil types, while S+NP+BC and P+NP+BC treatments showed synergistic effects of nanoparticles in stabilizing pH, though less pronounced than NPs or BC alone.

In this study, electrical conductivity (EC) increased slightly in both soil types (S-1 and S-2) without treatment, indicating a natural salinity rise (**Figure 2B**). The S+NP treatment reduced EC from 3.94 to 3.45 dS m^-1^ in S-1 and from 11.22 to 8.78 dS m^-1^ in S-2, showing that ZnO NPs alleviate salt stress. Similarly, the P+NP treatment decreased EC from 5.08 to 4.15 dS m^-1^ in S-1 and 10.95 to 8.92 dS m^-1^ in S-2. The S+BC treatment lowered EC from 3.7 to 3.45 dS m^-1^ in S-1 and from 11.3 to 9.57 dS m^-1^ in S-2, demonstrating biochar’s salt absorption capability. The P+BC treatment reduced EC from 4.52 to 4.35 dS m^-1^ in S-1 and from 10.01 to 9.37 dS m^-1^ in S-2. The S+NP+BC treatment decreased EC from 4.16 to 3.3 dS m^-1^ in S-1 and from 11.55 to 8.65 dS m^-1^ in S-2, while the P+NP+BC treatment reduced EC from 9.55 to 8.08 dS m^-1^ in S-2, highlighting the effectiveness of these combined treatments in reducing salinity (**Figure 2B**).

Analysis of organic matter (O.M) in saline soils showed significant increases, especially under moderate to high salinity conditions. In the control group, O.M decreased slightly in S-1 and S-2 (**Figure 2C**). Salinity reduces O.M by increasing the breakdown of carbon and altering bacterial communities. The application of biochar (S+BC) significantly boosted O.M levels, rising from 68 to 78 mg kg^-1^ in S-1 and 63 to 74 mg kg^-1^ in S-2. Nutrient combinations (S+NP+BC and P+NP) also enhanced levels, with P+NP+BC achieving the highest content at 86 mg kg^-1^ in S-1 and 81 mg kg^-1^ in S-2. These results highlight the effectiveness of biochar and its combinations in improving soil health under saline conditions.

### 3.3 Soil nitrogen, potassium, and phosphorus contents

**Figure 3:**
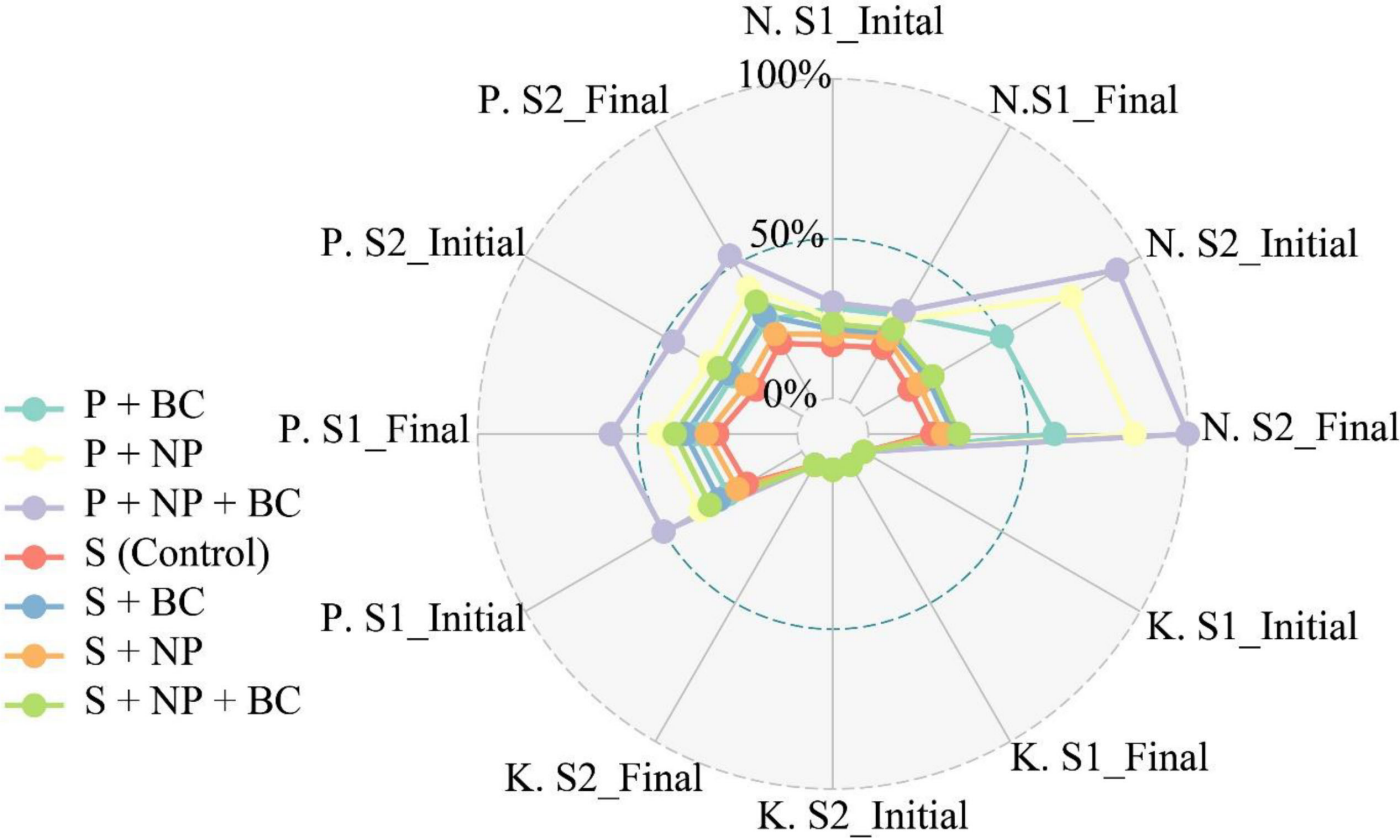
The effect of zinc oxide nanoparticles (NPs) and walnut biochar (BC) on soil macronutrients status under natural (S-1) and artificial saline soil (S-2). Soil total nitrogen (N), available phosphorous (P), and exchangeable potassium (K) were measured in natural saline soil (S-1) and artificially induced saline soil (S-2) following the application of NPs and BC treatments. The S-1 and S-2 final represent nutrient concentration determined after 15 days of treatment application.

The effects of treatments on soil nitrogen levels in S-1 and S-2 were evaluated. In the control group (S), nitrogen increased from 10.02 to 13.32 mg kg^-1^ in S-1 and from 3.33 to 6.4 mg·kg^-1^ in S-2 (**Figure 3**). The S+NP treatment significantly raised nitrogen content to 28.83 mg kg^-1^ in S-1 and 23.32 mg·kg^-1^ in S-2, indicating that ZnO NPs enhance nitrogen uptake under saline conditions. The P+NP treatment produced the most significant increases, particularly in S-1, where nitrogen content rose to 64.66 mg kg^-1^, and increased to 18.46 mg kg^-1^ in S-2. In the S+BC treatment, nitrogen contents rose in S-1 from 8.95 to 23.34 mg kg^-1^ and in S-2 from 2.93 to 23.06 mg kg^-1^. The P+BC treatment also increased nitrogen in S-1 from 9.52 to 25.37 mg kg-1 and in S-2 from 2.43 to 12.88 mg kg^-1^, suggesting biochar stabilizes nitrogen in saline soils. The S+NP+BC treatment elevated nitrogen in S-1 from 10.4 to 26.8 mg kg^-1^ and in S-2 from 2.36 to 24.19 mg kg^-1^, indicating the benefits of combining BC and ZnO NPs. In contrast, the P+NP+BC treatment resulted in a significant increase in S-1 (10.85 to 57.65 mg kg^-1^) but only minor growth in S-2 (1.73 to 10.9 mg kg^-1^), reflecting challenges in extreme salinity.

In the control group (S), potassium levels decreased in S-1 from 0.002 to 0.001 g kg^-1^ and in S-2 from 0.007 to 0.005 g kg^-1^, due to nutrient stabilization in saline conditions (**Figure 3**). In the S+NP treatment, potassium levels significantly increased in S-1 from 0.001 to 0.008 g kg^-1^ and in S-2 from 0.025 to 0.109 g kg^-1^. The P+NP treatment also showed increased, with S-1 rising from 0.005 to 0.042 g kg^-1^ and S-2 from 0.002 to 0.008 g kg^-1^, likely due to enhanced root function from ZnO NPs. The S+BC treatment increased potassium in S-1 from 0.009 to 0.069 g kg^-1^, attributed to biochar’s role in stabilizing potassium ions. In the P+BC treatment, potassium remained stable in S-1 but increased in S-2 from 0.004 to 0.024 g kg^-1^. The S+NP+BC treatment also increased potassium in S-1 from 0.014 to 0.033 g kg^-1^ and in S-2 from 0.002 to 0.025 g kg^-1^, showcasing the synergistic effects of biochar and ZnO NPs. Lastly, the P+NP+BC treatment raised potassium levels in S-1 from 0.006 to 0.008 g kg^-1^ and in S-2 from 0.015 to 0.025 g kg^-1^, indicating varied influences on potassium retention.

The analysis of phosphorus (P) levels highlights the effects of different treatments on soils S-1 and S-2 (**Figure 3**). In the control group, S-1 had P levels from 8.2 to 9.1 mg kg^-1^, while S-2 decreased slightly from 23 to 21.2 mg kg^-1^. Treatments like S+NP significantly increased P levels in both soils, with S-1 reaching 17.2 mg kg^-1^ and S-2–18 mg kg^-1^, due to enhanced uptake from ZnO NPs. The S+BC treatment resulted in a decline in P, reaching 19.7 mg kg^-1^ in S-1, likely due to the biochar’s ability to immobilize it. Meanwhile, P+BC increased levels in S-1 to 15.5 mg kg^-1^ and in S-2 to 16.1 mg kg^-1^, despite overall salinity reducing availability. The composite of S+NP+BC treatment stabilized levels in S-1 and significantly increased them in S-2 from 15.6 to 67.6 mg kg^-1^. Overall, non-salinized soil (S-1) showed much higher phosphorus availability compared to salinized soil.

### 3.4 Plant morphological traits and chlorophyll contents

Plants in the S-1 and S-2 soils showed a reduction in their root and shoot lengths, their fresh and dried weights. But the application of ZnO NPs and BC enhanced their vegetative growth. In S-1, the initial and final, the composite P + NP + BC increased the root and shoot lengths, reaching up to 18.40 cm and 26.50 cm, 21.22 cm and 27.85 cm as compared to the control treatment (**Table 1**). The shoot and root fresh and dry weight also moderately increased in the P + NP + BC among all the treatments. The same results were also observed in S-2, where the combined application of NP and BC increased the maize morphological traits. All soil stages exhibited a significant response (*p* < 0.05) from chlorophyll pigments to nitrogen treatments. When compared to the control, the concentrations of Chl-*a* and Chl-*b* in S-1 initially were significantly greater after the combination P + NP + BC treatment (16.31 and 11.25 mg g^-1^, respectively). The content of total chlorophyll (TC) rose to 27.54 mg g^-1^ as a result, suggesting improved photosynthetic capacity under combined administration. Chlorophyll buildup increased even more in the S-1 final. Compared to all other treatments, the P + NP + BC treatment produced noticeably greater levels of Chl-*a* (16.21 mg g^-1^), Chl-*b* (15.36 mg g^-1^), and TC (35.66 mg g^-1^). In comparison to the control, P + NP and P + BC also showed moderate but significant increases in chlorophyll pigments. Although total pigment concentrations decreased during S-2 beginning compared to S-1, the P + NP + BC treatment nevertheless significantly increased TC (32.36 mg g^-1^), Chl-*a* (16.31 mg g^-1^), and Chl-*b* (15.64 mg g^-1^) compared to the control and individual nutrient amendments. The strongest pigment reaction was seen by S-2 final. In comparison to all other treatments, the combined P + NP + BC treatment produced the highest levels of Chl-*a*, Chl-*b*, and TC (**Table 1**).

**Table 1:** Effect of walnut biochar (BC) and zinc oxide nanoparticles (ZnO NPs) on maize (*Zea mays*) morphological and photosynthetic traits under salinity stress.

Treatments		Root length (cm)	Shoot length (cm)	Root FW (g)	Root DW (g)	Shoot FW (g)	Shoot DW (g)	Chl-*a* (mg g^-1^)	Chl-*b* (mg g^-1^)	TC (mg g^-1^)
S (Control)	S-1 Initial	10.21 ± 1.02h	20.21 ± 2.15d	2.1 ± 0.15c	0.12 ± 0.01fgh	18.21 ± 2.15ef	10.11 ± 3.21e	10.16 ± 0.26h	8.65 ± 1.02g	21.36 ± 1.02i
P + NP		14.21 ± 1.11e	22.54 ± 2.16bc	1.01 ± 0.12f	0.15 ± 0.02e	18.22 ± 1.54ef	11.54 ± 1.66cd	11.89 ± 0.11g	10.25 ± 1.12e	22.36 ± 1.12h
P + BC		11.12 ± 1.25fg	21.55 ± 1.02c	1.11 ± 0.15f	0.13 ± 0.01fg	18.90 ± 3.21ef	11.20 ± 2.97cd	10.25 ± 0.64	10.31 ± 0.64e	21.36 ± 0.67i
P + NP +BC		18.40 ± 1.54b	26.50 ± 1.06ab	1.41 ± 0.36d	0.19 ± 0.01c	21.21 ± 3.45c	12.15 ± 2.64cb	16.31 ± 0.34b	11.25 ± 0.96d	27.54 ± 0.46e
S (Control)	S-1 Final	9.21 ± 1.25hi	21.22 ± 1.64c	3.1 ± 0.44b	0.14 ± 0.01f	19.55 ± 4.54d	12.11 ± 3.45cb	11.22 ± 0.99g	9.32 ± 0.67f	21.22 ± 0.67i
P + NP		12.40 ± 2.10f	23.97 ± 1.97b	3.22 ± 0.89b	1.01 ± 0.03ab	21.22 ± 2.0cc	13.54 ± 2.66ab	13.21 ± 0.87e	10.11 ± 0.74e	23.54 ± 0.99g
P + BC		11.10 ± 2.22fg	23.00 ± 1.48b	3.10 ± 0.47b	1.00 ± 0.05ab	20.58 ± 1.64cd	13.11 ± 2.97ab	12.22 ± 0.69f	10.64 ± 0.26e	24.32 ± 0.19f
P + NP +BC		21.22 ± 0.64a	27.85 ± 1.67a	4.11 ± 0.64a	1.05 ± 0.04ab	25.64 ± 1.95a	16.21 ± 6.54a	16.21 ± 0.94b	15.36 ± 0.34b	35.66 ± 0.48b
S (Control)	S-2 Initial	8.21 ± 0.67j	18.22 ± 1.49f	1.21 ± 0.88e	0.11 ± 0.09h	16.21 ± 1.58g	8.22 ± 3.99g	9.21 ± 0.67i	7.54 ± 0.44h	18.64 ± 0.56l
P + NP		9.11 ± 1.09hi	19.10 ± 1.99de	1.29 ± 0.31e	0.12 ± 0.07g	17.54 ± 5.54f	9.55 ± 3.48cf	10.31 ± 0.89h	8.54 ± 0.19g	20.87 ± 0.67j
P + BC		10.21 ± 1.05h	18.56 ± 1.47f	1.22 ± 0.45e	0.14 ± 0.03f	16.59 ± 3.24g	6.54 ± 2.40h	10.25 ± 0.54h	10.67 ± 0.94e	21.99 ± 0.22i
P + NP +BC		15.21 ± 1.11d	21.40 ± 0.25c	2.12 ± 0.99c	0.16 ± 0.04d	21.21 ± 1.64c	10.48 ± 2.11e	16.31 ± 0.67b	15.64 ± 0.56b	32.36 ± 0.49c
S (Control)	S-2 Final	7.21 ± 1.64k	17.22 ± 1.22g	1.01 ± 0.14f	0.10 ± 0.02hi	16.01 ± 2.88gh	8.12 ± 1.36g	9.99 ± 0.88i	8.22 ± 0.85g	19.54 ± 0.76k
P + NP		10.22 ± 1.87h	20.64 ± 0.67d	2.15 ± 0.34c	0.91 ± 0.03bc	18.59 ± 2.36ef	11.57 ± 1.97d	15.21 ± 0.36c	9.321 ± 0.66f	19.36 ± 0.39k
P + BC		11.21 ± 1.69fg	21.87 ± 0.99c	2.19 ± 0.85c	0.99 ± 0.01b	18.92 ± 1.54e	12.69 ± 1.55c	14.36 ± 0.54d	12.25 ± 0.21c	28.20 ± 0.44d
P + NP +BC		16.21 ± 1.66c	27.11 ± 0.48a	3.21 ± 0.94b	1.21 ± 0.01a	22.37 ± 6.14ab	15.98 ± 1.48b	19.64 ± 0.19a	17.65 ± 0.19a	40.25 ± 0.11a

Means (significance tested by LSD) followed by SE, the same letter(s) within each column are not significantly different among treatments (P ≤ 0.05).

Across both salinity levels (S-1 and S-2), net photosynthesis (Pn) and stomatal conductance (Gs) were declined from S-1 to S-2, with stronger reduction was observed in S-2, showing greater stomatal limitation and overall stress severity. Intercellular CO_2_ (Ci) was increased under salinity stress, especially the control indicating the CO_2_ assimilation was inhibited beyond the stomatal closure. Application of ZnO NPs and BC mitigated this effect by improving Pn and Gs and lowering Ci relative to control (**Supplementary Figure 1**). The combined application of ZnO+BC treatment produced the best recovery, suggesting better ion balance and antioxidant protection from Zn, improve water and nutrient retention and reduced Na^+^ stress from biochar, this improves the carbon fixation under salt stress.

### 3.5 Oxidative stress and antioxidant enzyme activities

**Figure 4:**
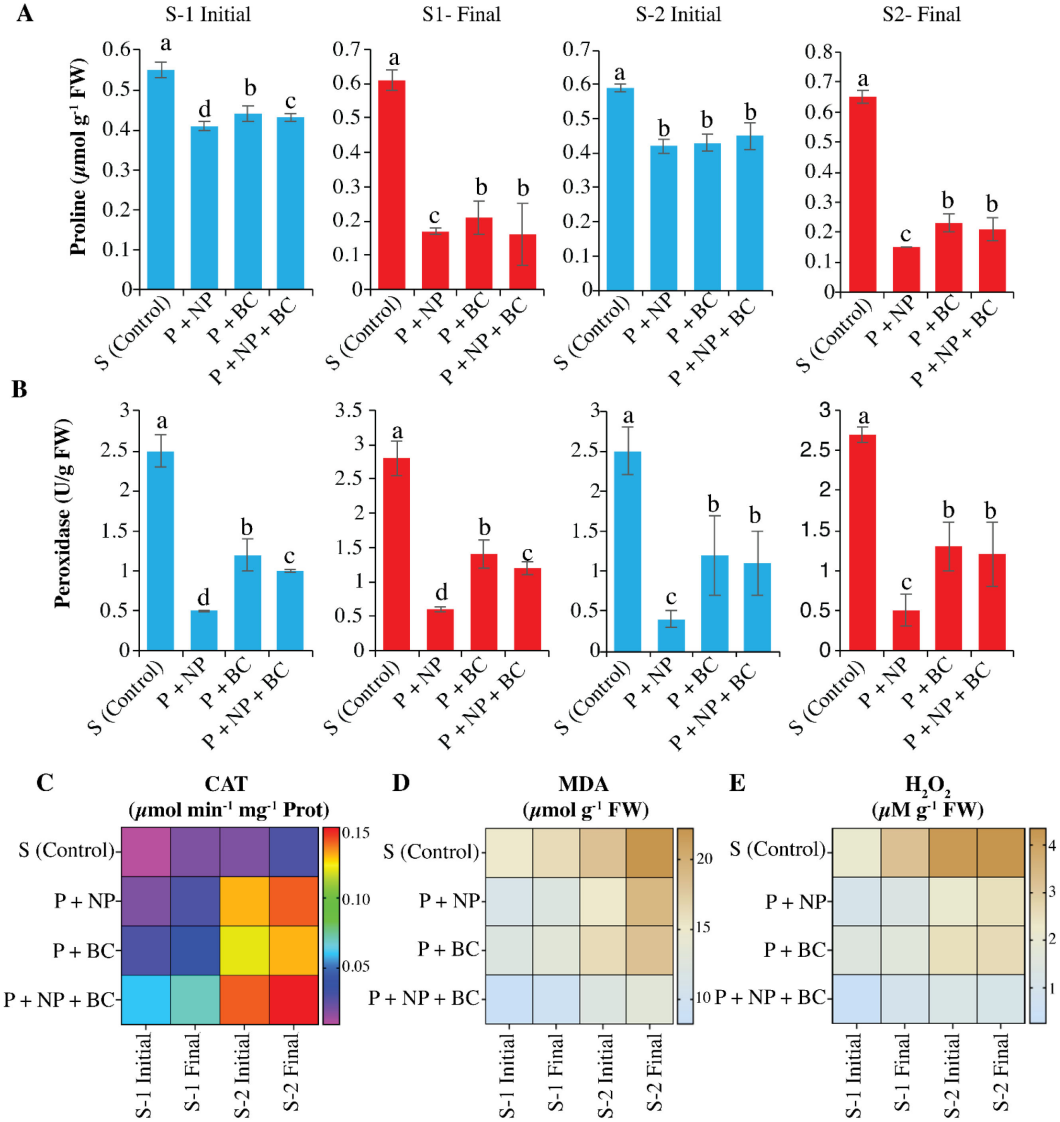
Effect of zinc oxide nanoparticles (NPs) and walnut biochar (BC) on oxidative and antioxidative responses of plants grown under natural and artificial salinity. Proline contents **(A)**, peroxide activity **(B)**, catalase activity **(C)**, malondialdehyde (MDA) contents **(D)**, and hydrogen peroxide (H_2_O_2_) concentration **(E)** in plants cultivated in natural (S-1) and artificial (S-2) saline soils. The S-1 and S-2 final represent the values after 15 days of treatment application. Error bars indicate the standard error (SE) of the mean (n = 3). Columns sharing the same lowercase letter are not significantly different (P ≤ 0.05).

Proline content in maize increased under salinity, confirming the activation of osmotic adjustment and stress protective metabolism in both S-1 and S-2 treatments. Proline, a vital osmoregulatory protectant, increased in S-1 (control) from 0.55 to 0.61 µmol g^-1^ FW and in S-2 from 0.59 to 0.65 µmol g^-1^ FW (**Figure 4A**). However, the response under the higher salinity level (S-2) showed comparatively less effective accumulation, suggesting that stress severity may have exceeded the plant’s capacity to maintain optimal compatible solute accumulation. Notably, application of ZnO NPs (P+NP) treatment markedly reduced proline concentration in both salinity regimes S-1 (from 0.41 to 0.17 µmol g^-1^ FW) and S-2 (from 0.42 to 0.15 µmol g^-1^ FW), indicating alleviation of salt-induced osmotic and oxidative stress. Conversely, ZnO NPs significantly enhanced proline content in plant leaves, with roots showing a remarkable 65.0% increase compared to controls. In the P+BC treatment, proline levels dropped moderately, from 0.44 to 0.21 µmol/g FW in S-1 and from 0.43 to 0.23 µmol g^-1^ FW in S-2. In the P+NP+BC treatment, proline levels decreased sharply in S-1 (from 0.43 to 0.16 µmol g^-1^ FW) and in S-2 (from 0.45 to 0.21 µmol g^-1^ FW). Overall, the incorporation of ZnO NPs effectively reduced proline levels across all treatments, making it the most effective method for stress alleviation, thereby minimizing the plant’s reliance on. In maize under salt stress, POD activity decreased in S-1 and S-2 due to prolonged stress (**Figure 4B**). ZnO NPs (P+NP) significantly reduced POD activity over 10 to 15 days, with the highest levels in ZnO NPs and the lowest in the control. The P+BC treatment also reduced activity under salinity stress. The P+NP+BC combination increased POD activity in both S-1 and S-2, indicating effective stimulation of antioxidative defenses in severe saline conditions.

The MDA and H_2_O_2_ contents also reduced in the combined application of the BC and ZnO NPs. Salinity increases the production of ROS and causes oxidative stress revealed by MDA and H_2_O_2_. Treatments of ZnO NPs and BC increased antioxidants, so that they reduced ROS and consequently MDA and H_2_O_2_ (**Figures 4D, E**). In this study, catalase activity increased slightly under control conditions but significantly decreased under high salinity. Control plants exhibited low catalase activity, while those treated with ZnO NPs showed a 69.7% increase (**Figure 4C**). The ZnO NPs raised catalase activity in control conditions before it declined under salinity stress, demonstrating enhanced activity particularly in saline environments. The P+BC biochar treatment reduced catalase activity under control conditions but showed a slight increase in saline conditions, indicating improved antioxidant. The P+NP+BC combination increased catalase levels under control conditions and stabilized activity at high salinity, indicating effective management of ROS.

**Figure 5:**
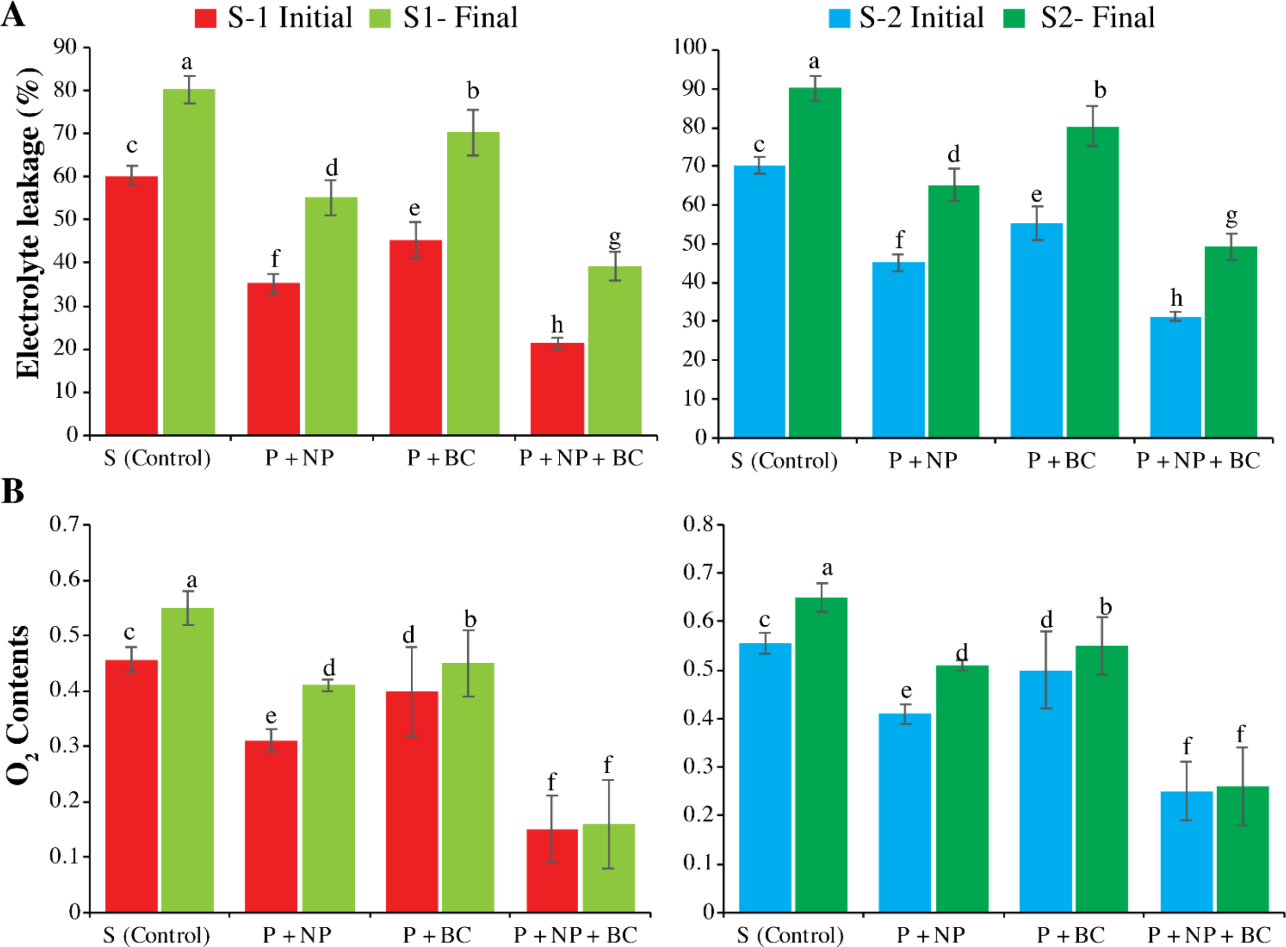
Effect of zinc oxide nanoparticles (NPs) and walnut biochar (BC) on membrane stability and reactive oxygen species production under salinity stress. Electrolyte leakage [EL, **(A)**] and dissolved oxygen [O_2_, **(B)**] concentration in plants grown in natural and artificial saline soil (S-1 and S-2). The S-1 and S-2 final represent the EL and O_2_ after 15 days of treatment application. Error bars indicate the standard error (SE) of the mean (n = 3). Columns sharing the same lowercase letter are not significantly different (P ≤ 0.05).

There was high electrolyte leakage in the control in both soils in S-1 and S-2 (60.22 and 80.21%). The sole application of biochar and NP reduces the leakage in both soils. The highest reduction in leakage was found in the combined application of the ZnO NPs and BC in both soils. The ZnO NPs and BC applications also reduce the O_2_ (0.15 and 0.16 µM g^-1^ FW) as compared to the control in both soils (0.456 and 0.65 µM g^-1^ FW) (**Figures 5A, B**).

## 4 Discussion

Different studies of nanoparticles reported that these are toxic for many plant species, but they can be utilized for abiotic and biotic stress tolerance, and they improve the plant growth (Zhou et al., 2020; Salam et al., 2022; Iqbal et al., 2025). However, the mechanistic function of ZnO NPs in mitigating the salt stress in maize remains inadequately understood. The environmentally friendly, non-toxic, stable, and cost-efficient green synthesis of zinc oxide nanoparticles (ZnO NPs) utilizing *Coriandrum sativum* has attracted considerable interest. In the current study, we also used ZnO NPs along with BC in soil to mitigate the salt stress in maize. The initial characterization of the synthesized ZnO NPs confirmed not only the successful production of the ZnO NPs but also their desirable properties (**Figure 1**). The NPs showed a unique surface plasmon resonance at 250–650 nm. Furthermore, the EDS analysis provided validation of the elemental composition, confirming the presence of key elements, including zinc, oxygen, and carbon, thereby affirming the formation of ZnO. FTIR spectroscopy bolstered this evidence by identifying the characteristic Zn-O bonds alongside vital functional groups (O-H, C-H, C=O), which play pivotal roles in ensuring ZnO NPs stability and dispersibility (Shaba et al., 2021).

The salinity stress increased H_2_O_2_ in plants, which may result in disrupted cellular functions and membrane damage through lipid peroxidation (Sun et al., 2023). Our study also observed that the application of ZnO NPs and BC promotes tolerance. Nanoform chitosan reduces the H_2_O_2_ and MDA levels, thereby promoting growth, increasing chlorophyll contents and enhancing plant metabolic processes (Cooper and Messina, 2023). During stress such as drought and salinity, Chitosan-NPs can improve antioxidants and osmolytes (Qasim et al., 2020). The present study observed elevated MDA levels in maize plants grown in saline soils, indicating a reduced stability of membrane molecules due to increased ROS accumulation. Our results showed that ZnO NPs and BC applications can reduce the salinity stress (**Figure 2**-**5**). This can be because BC can increase both the soil bulk density and porosity, which promotes the soil aggregation process. Increasing the soil porosity increased the leaching of soluble salts, which may reduce the soil salinity (Yahya et al., 2022). This is evident in the decrease in EC across treatments, particularly in the combined applications of S + NP + BC and P + NP + BC, which indicates successful salt leaching and enhanced plant resilience against salinity (Basit et al., 2022). The porous structure and high carbon content of BC likely contribute to its ability to sequester salts and enhance soil structure, a finding that is corroborated by previous meta-analyses (Wu et al., 2024). Additionally, treatments had a significant impact on the soil’s organic matter content and nutrient reservoir. While the control groups displayed a minor decrease in organic matter, presumably due to enzyme activity induced by salinity, the application of BC (S+BC, P+BC) and its combination with ZnO NPs (S+NP+BC, P+NP+BC) yielded substantial increases in organic matter (Kavitha et al., 2018). This highlights the crucial role that BC plays in enhancing soil health by providing stable carbon and promoting soil aggregation.

The BC+NPs application reduces ROS, MDA, and Electrolyte leakage, indicating that the treatment application mitigates salinity stress and may enhance plant growth. Another reason for mitigating salinity stress may be the high uptake of N, P, and K in BC+NPs applications. The published studies showed that such physiological maintenance occurs with reduced levels of oxidative stress (lower MDA and H_2_O_2_) and elevated capacities of antioxidant enzymes, enabling plants to shift carbon from stress repair to biomass accumulation (Rao et al., 2025). Salinity stress often induces osmotic and ionic imbalance in plants, leading to overproduction of ROS and disruption of nitrogen metabolism. In our study, the combined application of ZnO NPs and BC improved the soil N availability, which likely supported increased amino acid biosynthesis. Proline is recognized as a critical osmoregulatory protectant that typically accumulates under stress conditions. Proline biosynthesis is linked to nitrogen availability because it is primarily synthesized from glutamate, a nitrogen-containing amino acid. Treatment with ZnO NPs (P+NP, P+NP+BC) decreased proline levels in both soils (S-1 and S-2), indicating that ZnO NPs improved nitrogen assimilation and overall metabolic efficiency. Enhanced N uptake likely reduced the need for osmotic adjustment via proline accumulation, as plants could maintain turgor and osmotic balance more effectively under stress. Similarly, the addition of BC (P+BC) moderately lowered proline levels, suggesting improved soil nutrient availability and water holding capacity, which indirectly alleviates osmotic stress. The combined (P+NP+BC) treatment showed the most pronounced reduction in proline, highlighting the synergistic effect of ZnO NPs and BC in mitigating the salinity stress. This reduction signals a significant alleviation of osmotic stress (Faizan et al., 2021). It implies that the nanoparticles directly mitigate stress levels, thereby decreasing the plant’s necessity to produce excess proline. Additionally, the activities of antioxidant enzymes peroxidase and catalase were observed to be positively modulated. While prolonged stress led to diminished POD activity in control plants, the introduction of ZnO NPs and the combined treatment generally resulted in increased POD activity, portraying enhanced antioxidative defenses (Mahmoud et al., 2019). Catalase (CAT) activity, essential for the detoxification of hydrogen peroxide, also exhibited positive responses to ZnO NPs, particularly under saline conditions, emphasizing their role in bolstering the plant’s stress response and mitigating oxidative damage (Faizan et al., 2021). The diverse responses of these enzymes across treatments illustrate a complex interplay, yet overall, the amendments seem to augment the plants’ capacity to withstand saline challenges. The remarkable synergistic effects witnessed from the combined application of ZnO NPs and BC are especially noteworthy.

The notable reductions in pH, especially under extremely saline conditions (S-2), suggest that these amendments function to neutralize alkaline environments, thereby facilitating enhanced nutrient availability for plants. The levels of nitrogen, an essential nutrient for plant growth, significantly increased with the treatments involving both ZnO NPs and BC. This suggests that ZnO NPs may enhance nitrogen uptake even under saline conditions, while BC serves to stabilize nitrogen availability in the soil (Alabdallah and Alzahrani, 2020). Notably, potassium levels also showed substantial increases, particularly with treatments involving ZnO NPs and the combined approach, indicating improved potassium retention and uptake essential for osmotic regulation in plants under stress. In contrast, phosphorus availability exhibited varied responses; while ZnO NPs generally enhanced phosphorus levels, BC alone occasionally led to a decline, likely due to nutrient immobilization. However, the combined treatment, particularly in highly saline soil (S-2), demonstrated a remarkable increase in phosphorus availability, suggesting a synergistic effect that enhances nutrient accessibility. The physiological responses of the plants further highlight the benefits of the nano-bio composite. This can be shown in the morphological and chlorophyll results.

Salinity alters root-soil interactions and microbiological processes essential for nutrient cycling and root biological function (Ahmad et al., 2024). BC provides micro-habitants and labile carbon sources to boost microbial biomass and extracellular enzyme activities even in salt-stressed soils (Luo et al., 2025). Modified and nano-engineered forms of BC enhance microbe-mediated biochemical processes involved in C, N, and P cycling, thereby partially mitigating rhizosphere biochemical defects in salt-stressed plants and microbe-soil systems (Mustafa et al., 2026). ZnO NPs deliver bioavailable zinc to plant roots and microbe-metalloenzyme complexes, thereby enhancing the rhizosphere’s biochemical capacity to liberate nutrients (Hanif et al., 2024). In another study, nano-BC mixtures were also found to improve soil aggregate stability and pore connectivity in saline soil, which in turn promotes aeration and water flux to the rhizoplane (Yue et al., 2023). Taken together, it is clear that the observed values of increased nutrient uptake, lowered Na+ transport, and enhanced root growth in the combined treatment of ZnO-NPs + BC in our study are a result of rhizosphere processes. Mitigating salinity at the rhizosphere scale enables downstream physiological stability, thereby promoting sustainable growth and yield in maize. The combination strategy (P + NP + BC) hence maintains higher levels of chlorophyll and photosynthesis compared to saline conditions, enabling optimal carbohydrate absorption and allocation to the growing tissues. On an agricultural field scale, this synergistic use of ZnO-NPs and BC collectively alleviates plants’ stress-repair biology, redirecting plant physiological effort to sustained development and productivity for plant yield stability and agricultural sustainability in salt-affected soils. At the same time, the broader adoption of nano-BC strategies will require field-scale validation, long-term tracking of nanoparticle fate, and an integrative assessment of soil microbial functional sustainability to ensure environmental safety and agronomic sustainability.

## 5 Conclusion

**Figure 6:**
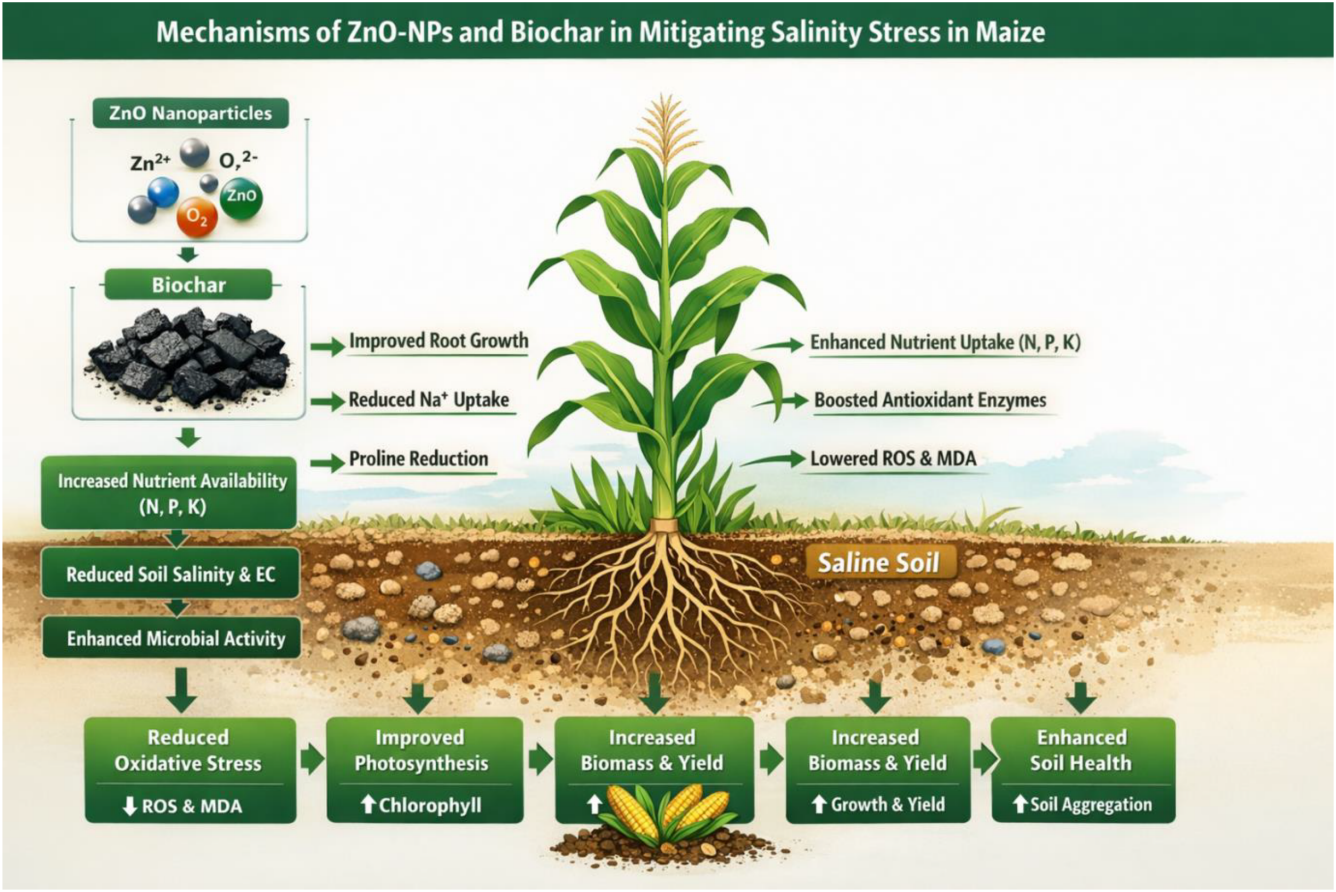
The effect of ZnO NPs (Nanoparticles) and BC (Biochar) on the maize growth under salinity stress.

In conclusion (**Figure 6**), this research provides strong evidence that green-synthesized ZnO NPs and walnut biochar, especially when applied together, offer a promising strategy for managing saline soils. They effectively reduce soil pH and electrical conductivity, enhance organic matter content, improve the availability of essential nutrients like nitrogen and potassium, and bolster the plant’s intrinsic defense mechanisms against salt stress. These findings significantly contribute to the development of sustainable agricultural practices for saline land reclamation, offering a viable pathway to enhance crop productivity in challenging environments. Future research could focus on optimizing the application rates of these composites, exploring their long-term effects on soil microbial communities, and evaluating their performance across a wider range of crop species and diverse saline soil types.

## Data Availability

The original contributions presented in the study are included in the article/**Supplementary Material**. Further inquiries can be directed to the corresponding authors.

## Supplementary material

Supplementary Figure 1 Effect of zinc oxide nanoparticles (ZnO NPs) and walnut biochar (BC) on gas-exchange traits under natural and artificial salinity conditions. Net photosynthetic rate (Pn, **(A)**), intercellular CO_2_ concentration (EC, **(B)**), and stomatal conductance (Gs, **(C)**) in natural saline soil (S-1) and artificially induced saline soil (S-2). The S-1 and S-2 final represent the values after 15 days of treatment application. Error bars indicate the standard error (SE) of the mean (n = 3). Columns sharing the same lowercase letter are not significantly different (P ≤ 0.05).
