# Supplementary material for: Green-synthesized zinc oxide nanoparticles and walnut biochar synergistically mitigate soil salinity and improve maize stress physiology
Source: Front Plant Sci. 2026 Mar 13;17:1788236. doi: 10.3389/fpls.2026.1788236 (PMC13022711; doi:10.3389/fpls.2026.1788236)
Supplement: Supplementary Figure 1 — Effect of zinc oxide nanoparticles (ZnO NPs) and walnut biochar (BC) on gas-exchange traits under natural and artificial salinity conditions. Net photosynthetic rate (Pn, (A)), intercellular CO2 concentration (EC, (B)), and stomatal conductance (Gs, (C)) in natural saline soil (S-1) and artificially induced saline soil (S-2). The S-1 and S-2 final represent the values after 15 days of treatment application. Error bars indicate the standard error (SE) of the mean (n = 3). Columns sharing the same lowercase letter are not significantly different (P ≤ 0.05). [file SupplementaryFile1.docx]

Green-Synthesized Zinc Oxide Nanoparticles and Walnut Biochar Synergistically Mitigate Soil Salinity and Improve Maize Stress Physiology

**Sangar Khan^1,2 †^, Jaweriah Naeem^3, †^, Aansa Rukya Saleem^3^*, Fiza Sarwar^3^, Asma Jamil, Habib Ullah^4,5^, Zepeng Rao ^4,5^*, Abubakr M Idris^6,7^ Waqar-Un Nisa^8^**

^1^Department of Geography and Spatial Information Techniques, Ningbo University, Ningbo 315211, China; (SK)

^2^Zhejiang-Germany Joint Laboratory on Remote Sensing of Coastal Ecosystem, Ningbo University, Ningbo 315211, China

^3^Department of Earth and Environmental Sciences, School of Engineering and Applied Science, Bahria University, Islamabad, Pakistan. (JN), (ARS), (FS)

^4^Department of Environmental Science, Zhejiang University, Hangzhou, China; (HU), (ZR)

^5^Innovation Center of Yangtze River Delta, Zhejiang University, Jiashan 311400, China.

^6^Department of Chemistry, College of Science, King Khalid University, Abha 62529, Saudi Arabia; [abubakr@yahoo.com](mailto:abubakr@yahoo.com) (AMI)

^7^Research Center for Advanced Materials Science (RCAMS), King Khalid University, Abha 62529, Saudi Arabia

^8^Center for Interdisciplinary Research in Basic Sciences (SA-CIRBS), International Islamic University, Islamabad, Pakistan; (WUN)

^†^ These authors contributed equally to this work.

*** Correspondence:**Corresponding Author
**Aansa Rukya Saleem** ; [arukya.buic@bahria.edu.pk](mailto:arukya.buic@bahria.edu.pk) (ARS); **Zepeng Rao** [raozepeng@zju.edu.cn](mailto:raozepeng@zju.edu.cn) (ZR)

Supplementary Material


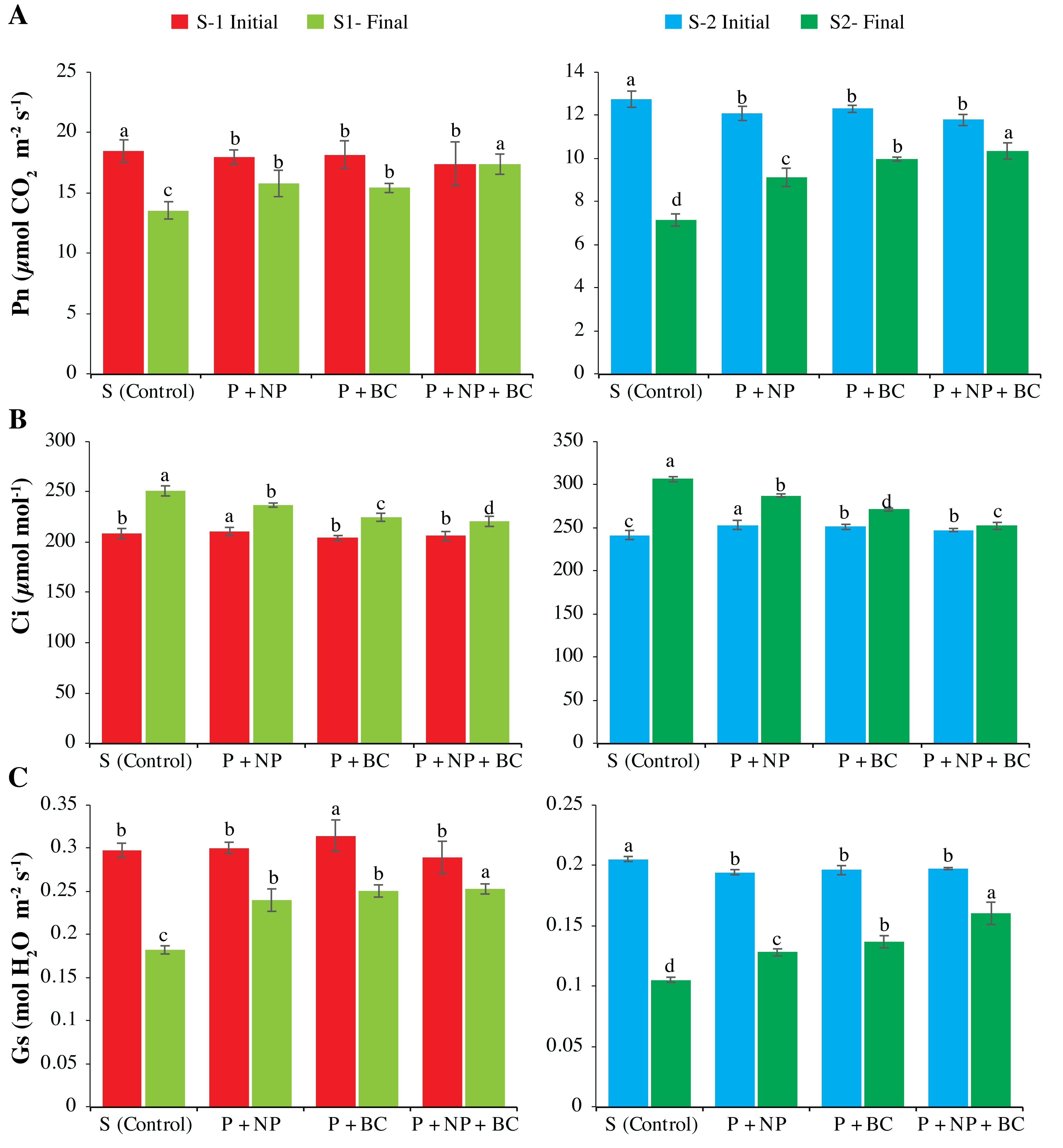


**Supplementary Figure 1.** Effect of zinc oxide nanoparticles (ZnO NPs) and walnut biochar (BC) on gas-exchange traits under natural and artificial salinity conditions. Net photosynthetic rate (Pn, **A**), intercellular CO_2_ concentration (EC, **B**), and stomatal conductance (Gs, **C**) in natural saline soil (S-1) and artificially induced saline soil (S-2). The S-1 and S-2 final represent the values after 15 days of treatment application. Error bars indicate the standard error (SE) of the mean (n = 3). Columns sharing the same lowercase letter are not significantly different (P ≤ 0.05).
